# Role of NLRP3 Inflammasome Inhibitors in Endothelial Dysfunction and Vascular Repair

**DOI:** 10.3390/antiox15070784

**Published:** 2026-06-24

**Authors:** Thangasrinivasan Samyuktha, Sridharan Yukta, Kumar Ganesan, Kunka Mohanram Ramkumar

**Affiliations:** 1 Department of Biotechnology, School of Bioengineering, SRM Institute of Science and Technology, Kattankulathur 603 203, Tamil Nadu, India; st8773@srmist.edu.in (T.S.); yuktasridharan@gmail.com (S.Y.); 2 School of Chinese Medicine, Li Ka Shing Faculty of Medicine, The University of Hong Kong, Hong Kong SAR, China; kumarg@hku.hk

**Keywords:** ED, NLRP3 inflammasome, inflammasome inhibitors, oxidative stress, cardiovascular diseases

## Abstract

Endothelial dysfunction (ED) is an early event in cardiovascular and metabolic diseases, including atherosclerosis, diabetes, and hypertension. Emerging evidence highlights the interplay between chronic inflammation and oxidative stress, collectively termed OxInflammation, as a major driver of vascular injury and impaired tissue repair. Among the key mediators of this response is the Nod like receptor family pyrin domain containing 3 (NLRP3) inflammasome, a multiprotein complex that promotes the release of inflammatory cytokines, including Interleukin 1β (IL-1β) and Interleukin-18 (IL-18), and induces gasdermin D-mediated pyroptotic cell death. Activation of NLRP3 disrupts endothelial function, reduces nitric oxide availability, and accelerates vascular inflammation and injury. This review discusses current evidence on pharmacological strategies targeting NLRP3 inflammasome signaling using both natural and synthetic inhibitors. Studies have shown that inhibiting NLRP3 can reduce inflammation and oxidative stress, preserve endothelial integrity, improve vascular function, and support tissue repair. Several NLRP3-targeting compounds have advanced into early-phase clinical trials, showing encouraging safety profiles and efficacy in individuals with cardiovascular risk factors. By integrating the emerging concept of OxInflammation with endothelial dysfunction, this review critically evaluates the therapeutic and translational potential of NLRP3 inflammasome inhibition in cardiovascular and metabolic disorders. Collectively, the available evidence supports NLRP3 as a promising therapeutic target for restoring endothelial homeostasis and promoting vascular repair. However, further clinical studies are needed to establish long-term efficacy, optimal dosing strategies, and appropriate patient selection criteria.

## 1. Introduction

Endothelial cells form the inner layer of blood vessels and create an important interface between circulating blood and vascular smooth muscle cells (VSMCs). These cells play a central role in vascular homeostasis through the regulation of vascular tone, vascular permeability, leucocyte adhesion and thrombosis [1]. Disruption of this regulatory balance leads to ED, which may arise from cellular injury or chronic stress [2,3]. ED is considered an early and central event in the development of atherosclerosis and other cardiovascular diseases [4,5]. It is characterized by elevated expression of pro-inflammatory cytokines, including monocyte chemoattractant protein-1 (MCP-1) and interleukin-8 (IL-8), enhanced leukocyte adhesion, and reduced vasodilation due to the loss of bioavailability of NO [6]. The development of ED is influenced by multiple well-established cardiovascular risk factors, including hypercholesterolemia [7], smoking [8], obesity, insulin resistance [9], high C-reactive protein (CRP) [10], and persistent low-grade infections [11]. In addition, the endothelial glycocalyx (EG), a carbohydrate-rich protective layer lining the luminal surface of endothelial cells and composed of glycosylphosphatidylinositol (GPI)-anchored proteins and other glycoconjugates, plays a critical role in maintaining vascular homeostasis by preventing leukocyte and platelet adhesion. EG damage has been associated with vascular complications, including those observed in fetal growth restriction (FGR) and vascular injury in diabetes [12]. Importantly, chronic inflammation is increasingly recognized as a key driving factor in the development and progression of ED.

Recent findings have highlighted the contribution of intracellular multiprotein complexes, known as inflammasomes, especially the NLRP3inflammasome, in the progression of vascular inflammation [13,14]. Cellular stress signals, including hyperglycemia, oxidative stress, and immune stimuli in the form of pathogen-associated molecular patterns (PAMPs) or damage-associated molecular patterns (DAMPs), can activate NLRP3 [15,16]. When the NLRP3 inflammasome is activated, it recruits and activates caspase-1, which subsequently cleaves the pro-inflammatory cytokines such as pro-IL-18 and pro-IL-1β into active forms [17]. These cytokines enhance the vascular inflammation and contribute to both structural and functional dysfunction of the endothelium [18].

Considering its central role in vascular inflammation and endothelial injury, the NLRP3 inflammasome has emerged as a promising therapeutic target in ED. Selective NLRP3 inhibitors, such as MCC950, have been shown to preserve endothelial nitric oxide synthase (eNOS) activity and maintain vascular endothelial (VE)-cadherin expression, thereby protecting endothelial barrier integrity and reducing vascular inflammation. Furthermore, accumulating evidence suggests that pharmacological inhibition of NLRP3 may interrupt the vicious cycle between oxidative stress and inflammation, a process increasingly recognized as OxInflammation, which contributes to the initiation and progression of cardiovascular and metabolic disorders. This review discusses the mechanistic role of the NLRP3 inflammasome in ED and evaluates the therapeutic potential of natural and synthetic NLRP3 inhibitors in mitigating OxInflammation, restoring endothelial function, and promoting vascular repair. While previous reviews have primarily focused on inflammasome biology or disease-specific inflammatory mechanisms, a comprehensive synthesis integrating OxInflammation, ED, tissue repair, and NLRP3-targeted therapies remains limited. Therefore, this review provides an updated overview of the molecular links between oxidative stress, inflammasome activation, and endothelial injury, while critically examining current therapeutic strategies, translational progress, and future challenges associated with NLRP3 inhibition.

### 1.1. ED in Cardiovascular and Metabolic Disorders

ED is a central pathological hallmark in various cardiovascular and metabolic conditions, such as atherosclerosis, hypertension, diabetes mellitus, Heart Failure with Preserved Ejection Fraction (HFpEF) and metabolic syndrome. Recent studies suggest that redox imbalance, NLRP3 inflammasome activation, and persistent inflammatory signaling play important roles in endothelial damage, vascular inflammation, and impaired vasodilatory function [19]. In addition, the role of inflammation in ED in HFpEF and mechanosensitive pathways such as Piezo1 and Transient Receptor potential Vanilloid-4 (TRPV4) in vascular inflammation and atherosclerosis related to hypertension has been identified [20,21]. Chronic low-grade inflammation (metaflammation) in metabolic syndrome further stimulates NLRP3 activation and vascular injury [22].

One of the most important events resulting from ED and vascular inflammation is acute myocardial infarction (AMI). The NLRP3 inflammasome plays important cell-specific roles in cardiomyocytes, endothelial cells, and immune cells during myocardial injury. Therefore, anti-inflammatory treatments that inhibit the activation of NLRP3, such as colchicine, have been investigated for their cardiac protective effects [23,24].

Another key context in which ED plays a role in disease progression is ischemia/reperfusion (I/R) injury. Reperfusion, as an important part of blood flow restoration, paradoxically produces oxidative stress, mitochondrial dysfunction, calcium overload and inflammatory activation [25,26]. The coronary circulation and vascular endothelium are especially susceptible to reperfusion-associated damage, and the preservation of the vascular endothelium is an important target of cardioprotection [27,28]. During reperfusion, the generation of excessive Reactive oxygen species (ROS) contributes to a decrease in NO bioavailability, increasing the vascular permeability and impairing the microvascular function, leading to adverse cardiovascular outcomes [29,30]. These findings are consistent with earlier reports describing the clinical relevance of reperfusion-induced injury [31].

The growing recognition of NLRP3-mediated inflammation as a key contributor to AMI and I/R injury has stimulated the development of novel therapeutic inhibitors. Experimental studies have demonstrated that pharmacological inhibition of the NLRP3 inflammasome mitigates myocardial I/R injury by promoting pro-survival pathways and preserving mitochondrial integrity and function [32,33]. Recently, new inhibitors like INF195, INF150 and INF200 have shown cardioprotective properties by inhibiting NLRP3-dependent inflammation and decreasing myocardial injury [34,35,36]. Moreover, NLRP3 inhibition can prevent human coronary endothelial cells from oxidative and lipotoxic stress, supporting its therapeutic value related to ED [37].

Aging is also a major contributor to ED and increased cardiovascular susceptibility. Chronic inflammation, oxidative stress, and NLRP3 inflammasome activation are associated with impaired vascular homeostasis and heightened vulnerability to ischemic injury during aging [38]. These changes help to explain the vascular dysfunction and poor outcomes seen in older people [39]. Furthermore, it was shown that activation of the NLRP3 inflammasome contributes to vascular senescence and diabetic vascular lesions, demonstrating the role of inflammaging in endothelial pathology [40]. Together, these results point to the NLRP3 inflammasome as a therapeutic target in ED in various disease states [41]. As a result, many natural, synthetic, and novel NLRP3 inhibitors have been explored as potential endothelial function-preserving therapies and as tools to help reduce cardiovascular damage.

### 1.2. Pathophysiology of Inflammasomes in ED

The NLRP3 inflammasome is a cytosolic multi-protein complex that acts as an important sensor of cellular stress and microbial signals. It is triggered by numerous signals, including intracellular bacterial and viral infections, and the activation of toll-like receptor (TLR2). Activation of the NLRP3 inflammasome occurs in two principal stages, namely, priming and activation (assembly) [42,43].

During the priming phase, the expression of NLRP3 and pro-IL-1β is transcriptionally upregulated by means of activation of the nuclear factor kappa B (NF-kB) pathway [44], in response to PAMPs or DAMPs [43]. The priming also involves post-translational modifications (PTMs) of NLRP3, including ubiquitination [45] and phosphorylation that facilitates NLRP3 to be competent in activation [42]. The second step involves the oligomerization and assembly of the NLRP3 inflammasome due to persistent stimulation of PAMPs or DAMPs [42]. These signals are normally initiated by pattern recognition receptors (PRRs) or inflammatory cytokines IL-1β and Tumor necrosis factor alpha (TNF-α) via their respective receptors [44]. Activated NLRP3 then interacts with an adaptor protein, ASC (apoptosis-associated speck-like protein containing a CARD), which facilitates the recruitment of Pro-caspase-1 to cleave into Active caspase-1 [46,47].

Active caspase-1 leads to the maturation of pro-inflammatory cytokines IL-1β and IL-18 and cleaves gasdermin-D (GSDMD). The N-terminal fragment of GSDMD forms pores in the cell membrane, enabling the release of inflammatory cytokines and inducing pyroptosis, a form of inflammatory programmed cell death distinct from apoptosis due to its membrane-disruptive and pro-inflammatory nature [48,49].

**Figure 1:**
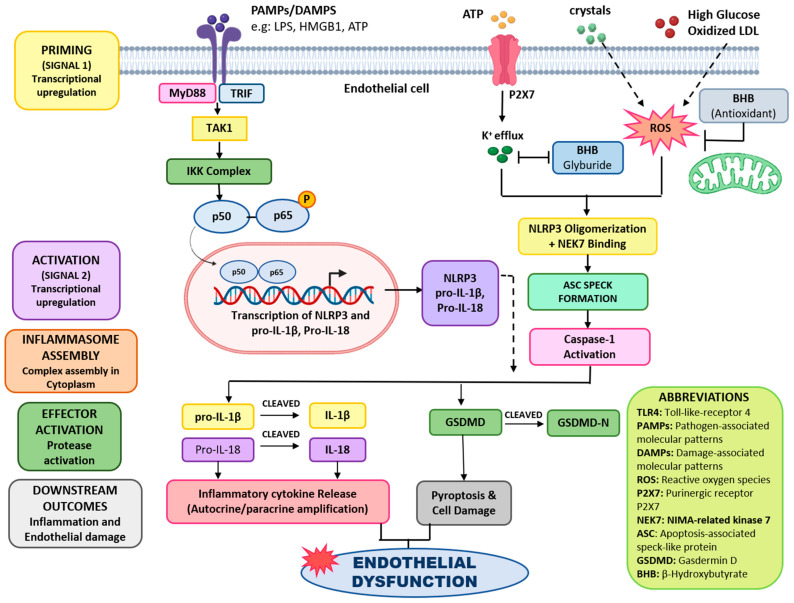
**Mechanistic Overview of NLRP3 Inflammasome Activation and Its Role in ED.** Priming signals induced by PAMPs/DAMPs activate NF-κB–dependent transcription of NLRP3 and pro-inflammatory cytokines, while activation signals such as ATP-mediated Potassium (K^+)^ efflux, ROS generation, and oxidized Low-density Lipoprotein (LDL) promote NLRP3 inflammasome assembly. Subsequent caspase-1 activation triggers IL-1β and IL-18 maturation, GSDMD-mediated pyroptosis, and inflammatory cytokine release, ultimately leading to endothelial injury and dysfunction. Potential inhibitory effects of β-hydroxybutyrate (BHB) and glyburide on NLRP3 activation are also indicated.

Within vascular tissue, these inflammatory reactions compromise endothelial integrity and induce pathological remodeling. Recent research shows a strong association between activation of the NLRP3 inflammasome and cardiovascular diseases, mainly by mediation of ED [50]. Several pathogenic triggers, including oxidative stress, lysosomal rupture, release of cathepsin B, and increased production of ROS, can trigger NLRP3 in endothelial cells [51]. These processes lead to endothelial barrier disruption, increased vascular permeability, and enhanced pro-inflammatory signaling. Persistent activation of the NLRP3 inflammasome triggers a sequence of inflammatory reactions and oxidative stress, which eventually results in vascular dysfunction [52]. The NLRP3 inflammasome represents a promising therapeutic target for preventing endothelial damage [53]. A schematic representation of the NLRP3 pathway is depicted in Figure 1.

### 1.3. OxInflammation as a Driver of ED

OxInflammation refers to the interconnection between oxidative stress and inflammation, which can both trigger ED and are strong factors in cardiovascular disease. The NLRP3 inflammasome plays a central role in these two processes [54,55]. Excessive ROS, especially mitochondrial ROS (mtROS), can induce the dissociation of thioredoxin-interacting protein (TXNIP) from thioredoxin, so that it can bind and activate NLRP3 [56]. Furthermore, ROS-mediated induction of NF-κB is needed for the priming of NLRP3 expression [57]. K^+^ efflux and mitochondrial dysfunction are subsequent triggers that promote inflammasome assembly and activation of caspase-1 [58,59]. Importantly, the pro-inflammatory cytokines IL-1β and IL-18, generated downstream of NLRP3 activation, exacerbate inflammatory responses and vascular injury, further promoting ROS production through mitochondrial dysfunction [60].

**Figure 2:**
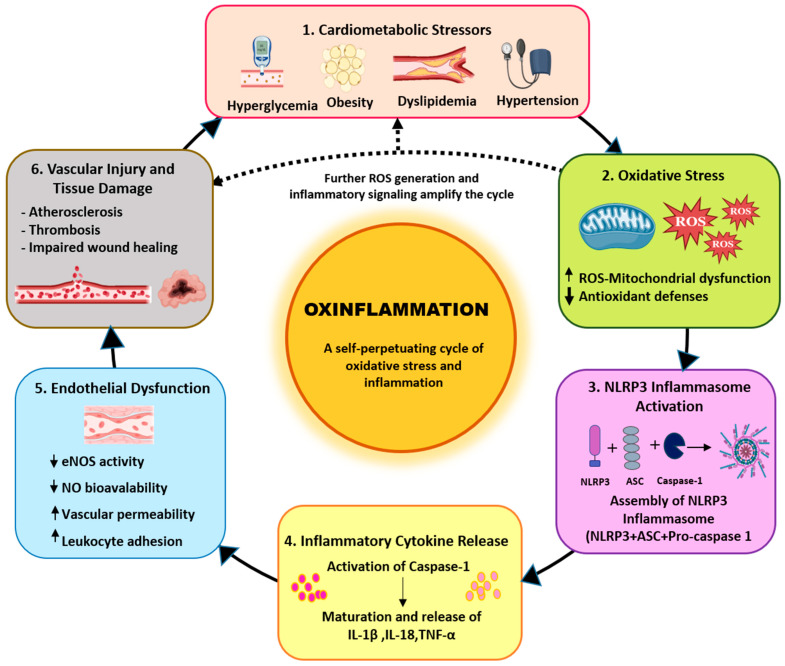
**Schematic illustration of the oxinflammation cycle linking cardiometabolic stressors to vascular injury.** Cardiometabolic risk factors, including hyperglycemia, obesity, dyslipidemia, and hypertension, promote oxidative stress and mitochondrial dysfunction, leading to NLRP3 inflammasome activation and the release of pro-inflammatory cytokines. These events induce ED and vascular damage, which further amplify oxidative stress and inflammation, establishing a self-perpetuating cycle of oxinflammation.

This leads to activation of oxidant-generating enzymes, which is a positive feedback loop between oxidative stress and inflammation. Persistent OxInflammation is responsible for endothelial injury through NO depletion, increased vascular permeability and enhanced expression of adhesion molecules [61,62]. As oxidative and inflammatory damage continues, it eventually leads to ED and cardiovascular diseases [63]. Thus, NLRP3 inhibitors may offer bifunctional therapeutic actions, acting both as a suppressor of oxidative stress and inflammatory signaling, therefore interrupting the OxInflammation cycle and maintaining endothelial integrity. A schematic representation of the OxInflammation cycle linking cardiometabolic stressors to vascular injury is depicted in Figure 2.

## 2. Literature Search Strategy

This article is a narrative review intended to provide an overview of current advances in NLRP3 inflammasome inhibitors and their therapeutic potential in endothelial dysfunction. The literature was collected through searches of electronic databases, including PubMed, Scopus, and Google Scholar, using combinations of keywords such as “NLRP3 inflammasome”, “endothelial dysfunction”, “OxInflammation”, “oxidative stress”, “cardiovascular disease”, “metabolic disease”, “vascular inflammation”, and “NLRP3 inhibitors”. Relevant original research articles, review articles, and preclinical and clinical studies published in English were considered. Additional references were identified through citation tracking of selected articles. The included studies were selected based on their relevance to the scope of the review rather than a predefined systematic review protocol.

## 3. Inflammasome Inhibitors as Therapeutic Agents

The therapeutic intervention targeting the NLRP3 inflammasome consists of multiple approaches that discuss at different stages of its activation and downstream signaling pathways. Such strategies involve inhibition of the NLRP3 itself, the assembly of the inflammasome complex, upstream signals, caspase-1 activation and GSDMD, a pore-forming protein, cleavage that leads to pyroptosis and the release of pro-inflammatory cytokines like IL-1β and IL-18. Molecular interventions encompass blocking NLRP3 complex assembly, inhibiting purinergic receptor (P2X7) to inhibit K^+^ efflux, and the use of ROS scavengers to alleviate oxidative stress, one of the main factors triggering inflammasome activation. They focus on the vital points in the inflammatory process that are controlled by inflammasome signaling to suppress the inflammatory cascade [64,65,66,67]. A wide range of NLRP3 inhibitors has been identified, which can broadly be classified into direct inhibitors, natural compounds, and synthetic inhibitors. These classes exhibit distinct mechanisms of action and offer diverse therapeutic opportunities for modulating inflammation and preventing endothelial injury.

## 4. Direct NLRP3 Inhibitors

Direct NLRP3 inhibitors are a potential class of therapeutic compounds that inhibit inflammasome activation directly via the NLRP3 protein by blocking its assembly and activation. Direct inhibitors act directly on key molecular events needed for inflammasome formation to block caspase-1 activation and pro-inflammatory cytokine maturation (e.g., IL-1β and IL-18). These inhibitors have shown significant potential in reducing OxInflammation, maintaining endothelial function and reducing tissue injury in cardiovascular and metabolic diseases by selectively inhibiting NLRP3. In this review, the direct NLRP3 inhibitors mentioned are the natural compound Oridonin and synthetic inhibitors like MCC950, CY-09, OLT1177, and 3,4-Methylenedioxy-β-nitrostyrene (MNS).

### 4.1. Natural Direct NLRP3 Inhibitors

#### Oridonin

Oridonin (Ori) is a bioactive diterpenoid derived from *Rabdosia rubescens* that has been researched extensively as an anti-inflammatory and cytoprotectant. Ori has the potential to suppress NF-kB, which fuels NLRP3 [68], and has also been shown to have neuroprotective effects apart from vascular diseases [69]. Mechanistic studies have shown that Ori covalently binds to the cysteine 279 residue of NLRP3, thereby disrupting its interaction with NIMA-related Kinase 7 (NEK7) and preventing assembly of the inflammasome complex. This further inhibits caspase-1 activation and IL-1β release in LPS-stimulated bone marrow-derived macrophages (BMDMs) and human PBMCs, without affecting the TNF-α expression [70]. Additionally, C57BL/6 mice with myocardial infarction treated with Oridonin showed reduced infarct size, fibrosis, and NLRP3/IL-1β/IL-18 expression, proving Oridonin protects via NLRP3 inflammasome inhibition [71]. Similarly, oridonin-treated ApoE^−/−^ mice with atherosclerosis exhibited reduced oxidative stress, NLRP3 activation, foam cell formation, macrophage infiltration, and plaque progression, mediated through suppression of NLRP3 and stabilization of Nuclear erythroid related factor 2 (Nrf2) [72].

### 4.2. Synthetic Direct NLRP3 Inhibitors

#### 4.2.1. MCC950

MCC950 is a synthetic small-molecule NLRP3 inflammasome inhibitor that has been demonstrated to have considerable evidence in both *in vitro* and *in vivo* settings. This prevents both canonical and non-canonical activation of NLRP3, thereby inhibiting caspase-1, ASC oligomerization and IL-1β synthesis via NEK7-NLRP3 interactions as seen in ASC-YFP imaging and GSTO1-binding [73]. Notably, it does not affect the NLRC4-mediated pyroptosis and, therefore, is selective in relation to ASC-dependent pathways. *In vitro* tests with BMDMs, human brain endothelial cells, and retinal endothelial cells have demonstrated that MCC950 suppressed caspase-1 (p20) and GSDMD cleavage, decreased MMP9 and other inflammatory mediators and that MCC950 preserved endothelial cell function in the presence of high-glucose stress or oxidative stress [74]. MCC950 has also been demonstrated to inhibit IL-1β, increase re-endothelialization, and decrease neointimal hyperplasia in vascular graft mouse models, which is better than the traditional agents like paclitaxel and sirolimus [18]. In diabetic rat models induced by a high-fat diet and streptozotocin, as well as in Human umbilical vein endothelial cells (HUVECs) treated with high glucose and high fat, MCC950 restored vascular health by inhibiting NLRP3 activity and mitigating the effects of adiponectin or peroxynitrite in the context of NLRP3 overexpression [75]. In addition, MCC950 stimulated neurovascular remodeling, cognition via Brain-Derived Neurotrophic factor (BDNF) signaling [76] and retinal endothelial apoptosis prevention through NEK7-NLRP3 interaction, which are translationally relevant to the proliferative diabetic retinopathy [77]. Collectively, these data indicate that MCC950 can be used as a therapeutic agent in the treatment of ED in various inflammatory and metabolic disorders.

#### 4.2.2. CY-09

CY-09 is a small-molecule direct NLRP3 inhibitor derived from CFTR(inh)-172 that has demonstrated significant anti-inflammatory activity in both *in vitro* and *in vivo* models. Mechanistically, CY-09 directly binds to the ATP-binding motif within the NACHT domain of NLRP3, thereby suppressing ATPase activity and preventing inflammasome activation. This direct interaction inhibits NLRP3 oligomerization, caspase-1 activation, and the maturation of pro-inflammatory cytokines [64].

In addition, CY-09 is reported to induce a cytoprotective effect in the hypoxia/reoxygenation-stressed cardiac microvascular endothelial cells and, therefore, reduce myocardial injury in the I/R rat models by inhibiting the NLRP3/caspase-1 signaling pathway [78]. CY-09 has also shown its potential over an atherosclerosis model, which is another hallmark of endothelial dysfunction. Studies on ApoE knockout mice demonstrated that CY-09 is effective in preventing atherosclerosis by enhancing the functionality of the valves of the aorta and diminishing the aortic valve calcification, linked to a decrease in IL-6 and TNF-α levels [79]. In addition to these more traditional disease models, CY-09 was also used to develop InflammaProbe-1, a fluorescent imaging probe that retained NLRP3-inhibitory activity in addition to anti-inflammatory behaviour in BMDMs and in a mouse model of age-related macular degeneration utilizing laser-induced choroidal neovascularization [80].

#### 4.2.3. OLT1177

OLT1177, a β-sulfonyl nitrile small-molecule inhibitor, is a consistent inhibitor of NLRP3 inflammasome activation. Trimethylamine N-oxide (TMAO)-induced inflammasome activation was inhibited by OLT1177 in Endothelioma mouse aortic E (EOMA) cells, and reduced NLRP3, ASC, and caspase-1 levels were obtained, with no impairment of endothelial barrier function [81]. The same protective effect was also reflected in the human microvascular endothelial cells (HMEC-1), in which OLT1177 reversed Envelope Protein Domain III (EIII)-induced pyroptosis, and provided defence against dengue-related vascular injury [82]. In animal models, mice with AMI treated early had a smaller infarct size with higher ventricular systolic function due to inhibition of NLRP3 activation [83]. Experiments with monocytes in Cryopyrin-associated periodic syndrome (CAPS) patients, as well as with macrophages and neutrophils, in human-derived systems revealed a significant decrease in IL-1β, IL-18, and caspase-1 activity after OLT1177 exposure [84]. Taken together, these findings highlight the therapeutic potential of OLT1177 as a versatile agent with applicability across a broad spectrum of cardiovascular, infectious, and autoinflammatory conditions.

#### 4.2.4. 3,4-Methylenedioxy-β-nitrostyrene (MNS)

MNS is another selective small-molecule NLRP3 inflammasome inhibitor that has shown a consistent anti-inflammatory effect in preclinical trials. Mechanistically, MNS works by blocking ASC oligomerization and thus fails in NLRP3 inflammasome assembly, which in turn inhibits activation of caspase-1 and maturation of IL-1β and IL-18. Importantly, MNS selectively inhibits NLRP3, while having a limited effect on other inflammasome complexes such as NLRC4 and AIM2 [85]. In animal models, MNS exhibited reno-protective activity in a rat model of I/R injury through the suppression of tubular injury and inhibition of PANoptosis, a combined process of pyroptosis, apoptosis, and necroptosis, by inhibiting NLRP3 [86].

## 5. Indirect Regulation of Upstream Signaling Pathways

Unlike direct NLRP3 inhibitors, these compounds suppress inflammasome activation by targeting upstream signaling events that contribute to NLRP3 priming and activation. Key upstream regulatory mechanisms include oxidative stress, ROS generation, NF-κB signaling, mitochondrial dysfunction, TXNIP activation, and ionic flux disturbances. By modulating these pathways, upstream regulators indirectly inhibit NLRP3 inflammasome activation, thereby reducing caspase-1 activation, pro-inflammatory cytokine release, ED, and OxInflammation. Several natural and synthetic compounds have demonstrated protective effects through these mechanisms and are discussed below.

### 5.1. Natural Regulators of Upstream NLRP3 Signaling

#### 5.1.1. Berberine

Berberine (BBR), an alkaloid phytochemical derived from *Berberis silvatica*, is well known to inhibit the NLRP3 inflammasome and prevent vascular damage associated with metabolic diseases. BBR prevented palmitate-induced endothelial injury by promoting eNOS expression and reducing NADPH oxidase 4 (NOX4) activity in HUVECs [87]. Similarly, the inhibition of NLRP3-mediated endothelial damage in lipopolysaccharide (LPS)-induced vascular injury of C57BL/6J male mice by BBR was also observed, which was further confirmed by the inhibition of inflammasome activation in LPS-ATP-challenged mouse microvascular endothelial cells (MECs) [88]. In comparison, a derivative, 13-methylberberine (13-MB), displayed protective effects in the HUVECs by inhibiting the NLRP3 inflammasome activity via an autophagy-mediated pathway under the influence of hydrogen peroxide-mediated oxidative stress, thus revealing a different mechanism of action to that of its parent compound [89].

#### 5.1.2. Curcumin

Curcumin is a bioactive polyphenol that has gained interest as a therapeutic agent because of its strong anti-inflammatory effects, especially in those metabolic pathologies like diabetic nephropathy. As an anti-inflammatory compound, curcumin displayed its potential against the NLRP3 inflammasome in renal injury, splenomegaly and diabetes [90,91,92]. Curcumin enhanced endothelial function and reduced atherosclerosis-associated endothelial and inflammatory responses by inhibiting the activation of the NLRP3/ASC inflammasome and inflammatory cytokines production in ApoE^−/−^ atherosclerotic mice and human aortic endothelial cells (HAECs) [93]. Similarly, in HUVECs, curcumin reduced NLRP3 inflammasome activation, alleviated pyroptosis, and enhanced endothelial cell function [92]. Mechanistic studies have also shown that curcumin selectively inhibits NLRP3 inflammasome activation by blocking potassium efflux, preserving mitochondrial integrity, and preventing ASC oligomerization and speck formation. With this mechanism, curcumin was shown to decrease secretion of IL-1β and to improve insulin sensitivity in high-fat diet–fed wild-type C57BL/6 mice, but not in Nlrp3^−/−^ mice, underlining the specificity of the protection by curcumin through inhibition of NLRP3 [94].

#### 5.1.3. Resveratrol

Resveratrol (RSV) is a natural polyphenol well-represented in grapes and pines and has been known to possess extensive biological activity, such as anti-oxidant, anti-inflammatory, and cytoprotective properties. In systemic inflammation, RSV treatment rescued LPS-induced damage in Sprague-Dawley rats by inhibiting TLR4 signaling, turning off pro-inflammatory mediators, increasing Interleukin-10 (IL-10) levels, and blocking the activation of NF-kB and Mitogen-Activated protein kinase MAPK in both liver and lung tissues [95]. Additionally, multiple studies have shown that it can ameliorate endothelial dysfunction by modulating NLRP3 inflammasome signaling. Resveratrol (RSV) suppresses endothelial pyroptosis by regulating the SIRT1/p66Shc/NLRP3 pathway, thereby restoring endothelial integrity in HUVECs exposed to palmitic acid and in mice fed a high-fat diet [96]. Furthermore, RSV protected mice in a retinal I/R model by inhibiting the activation of the NLRP3 inflammasome, pyroptosis, and oxidative stress, as well as enhancing the antioxidant defense through Kelch-like ECH associated protein-1 (Keap1)/Nrf2, which consequently upregulated Heme oxygenase-1 (HO-1) expression [97,98]. Additionally, in diabetic mice, it was demonstrated that RSV has beneficial effects on endothelial function by reducing vascular oxidative stress and inflammation by blocking the action of TNF-α [99]. Overall, these results suggest that RSV is a potential NLRP3-targeted therapeutic agent to reduce ED and vascular damage.

#### 5.1.4. Naringenin

Naringenin (NG) is a naturally occurring, low-toxicity flavonoid with notable anti-inflammatory, antioxidant, and cardiometabolic protective properties. NG inhibited endothelial inflammation in TNF-α-treated rat intestinal microvascular endothelial cells (RIMVECs) *in vitro* by blocking the hypersecretion of IL-6 and the activation of the TLR4/NF-κB/NLRP3 and Myosin Light Chain Kinase (MLCK)/phosphorylated Myosin Light Chain (p-MLC) signaling pathways [100]. Moreover, NG protected against myocardial dysfunction, oxidative stress, oxidative stress-related organ dysfunction and NLRP3 activation through miRNA-208a signaling pathway *in vivo* rats with myocardial infarction induced with isoproterenol [101].

#### 5.1.5. β-Hydroxybutyrate (BHB)

The ketone body produced during fasting or ketogenic diets, β-hydroxybutyrate (BHB), has been observed to be a strong NLRP3 inflammasome inhibitor. BHB has been reported to inhibit inflammasome activation, thereby preventing IL-1β secretion in several models [65]. BHB administration decreased the activity of retinal NLRP3 inflammasome and endoplasmic reticulum (ER) stress markers in C57BL/6J diabetic mice, indicating its therapeutic value in the prevention of diabetes-induced inflammation in the retina [102]. Along with this, its structural isomer, 3-hydroxybutyrate (3-HB), was demonstrated to inhibit atherosclerosis and IL-1β synthesis by blocking NLRP3 inflammasome activation in apoE^−/−^ mice and BMDMs, highlighting the translational promise of ketone bodies in chronic inflammatory disorders [103].

### 5.2. Synthetic Regulators of Upstream NLRP3 Signaling

#### Glyburide

Glyburide, a sulfonylurea-class antidiabetic drug, has also been reported to indirectly inhibit the NLRP3 inflammasome. Glyburide suppressed the release of IL-1β and activation of caspase-1 but did not degrade cell viability or clearance of microbes in BMDMs and peripheral blood mononuclear cells (PBMCs) of FCAS patients [66]. Glyburide (glibenclamide) inhibited NALP3 signaling without involving the inflammasome, thereby improving endothelial function. It has been demonstrated that it restores the expression of p-eNOS in high-salt-treated mouse aortic endothelial cells (MAECs) *in vitro* and reduces ED in high-salt-fed mice [104]. Additionally, *in vitro* studies showed that glibenclamide was able to inhibit NLRP3 activation, to increase NO, to restore VE-cadherin expression and to reduce oxidative stress in endothelial cells treated with preeclamptic plasma, thus preventing endothelial cell damage [105].

## 6. Inhibition of Downstream Inflammatory Effector Mechanisms

In addition to direct inhibitors of NLRP3 or upstream regulators of its signaling pathway, certain compounds also act by targeting downstream inflammatory mediators activated following inflammasome assembly. The downstream effect targets are the activation of caspase-1, maturation of IL-1β and IL-18, pyroptotic cell death, and NF-κB-mediated inflammatory response. These agents inhibit these effector pathways and thus help preserve endothelial function and repair by decreasing endothelial inflammation, vascular injury, and OxInflammation-associated tissue damage.

### 6.1. JC124

JC124 is a small molecule selective NLRP3 inhibitor that has been reported for anti-inflammatory and vasculoprotective properties. In endothelial models, JC124 prevented the activation of the NLRP3 inflammasome and maintained the integrity of tight junctions, thus protecting against trimethylamine N-oxide (TMAO) induced endothelial injury [106]. The results indicate JC124 is a potential therapeutic candidate for reducing ED.

### 6.2. Parthenolide

Parthenolide (PTL), a sesquiterpene lactone isolated from *Tanacetum parthenium*, is a well-characterised anti-inflammatory molecule with direct actions on the inflammasome. Earlier studies by Juliana et al. also showed that PTL did not need to prime macrophages to encourage the activity of various inflammasome complexes, including NLRP3, but instead directly suppressed caspase-1, which activates a variety of inflammasomes [107]. Notably, its endothelial applicability was investigated in vascular dysfunction during sepsis; in the LPS-induced septic rats, as well as in LPS-stimulated endothelial cells. PTL enhanced survival, mitochondrial integrity, and endothelial barrier function. In addition to that, these effects were enhanced further by nano-formulated PTL (N-PTL), highlighting its translational potential in endothelial injury and coagulopathy in sepsis [108].

**Figure 3:**
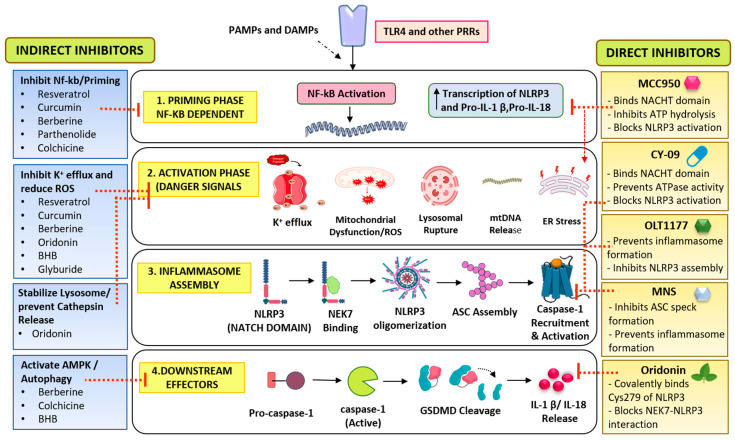
**Schematic representation of the direct and indirect modes of NLRP3 inhibition by both the natural and synthetic inhibitors.** The figure highlights compounds that suppress NLRP3 inflammasome activation by modulating the priming phase, activation signals, inflammasome assembly, or downstream effector pathways, thereby reducing caspase-1 activation and pro-inflammatory cytokine release. (Red dashed lines indicates inhibition).

A summary of natural and synthetic NLRP3 inhibitors, their experimental models, and key outcomes in ED is presented in Table 1 and Table 2, respectively. A schematic representation of the direct and indirect modes of NLRP3 inhibition by both the natural and synthetic inhibitors is depicted in Figure 3.

## 7. Clinical Evidence and Therapeutic Potential

### 7.1. Current Clinical Evidence

One of the promising therapeutic options in the prevention of ED, which is a major cause of cardiovascular diseases, is NLRP3 inflammasome inhibition. Promising findings of *in vitro* and *in vivo* research have become the basis of translational developments. Indicatively, Sinapine Thiocyanate (ST) has been demonstrated to inhibit NLRP3 activation in HUVECs stimulated with angiotensin II and in spontaneously hypertensive rats (SHRs), thus resulting in functional improvement of the endothelia, based on increased generation of and decreased endothelin-1 levels [109]. Likewise, a selective NLRP3 inhibitor, MCC950, had protective effects in several models: in HUVECs exposed to hypoxic stress that models preeclampsia and in reduced uterine perfusion pressure (RUPP) rats, where it decreased amounts of oxidative stress and enhanced endothelial-dependent vasodilation [114]; in diabetic rats, where it rescued endothelial endothelial function. These findings underscore the translational significance of targeting NLRP3 for vascular protection across a range of pathological conditions.

Upon this preclinical success, a few selected NLRP3 inhibitors have advanced to clinical development. DFV890 was well-tolerated and dose-limiting toxicities were absent in healthy volunteers in a Phase I trial, and there was evidence of target engagement [115]. Moreover, Phase II trials of Dapansutrile (OLT1177) showed a decrease in systemic inflammation and better vascular outcomes in patients with cardiovascular risk factors [116]. The clinical development status and translational progress of lead NLRP3 inhibitors are presented in Table 3. Collectively, these data indicate a clear progression from mechanistic studies to early clinical evaluation, suggesting that NLRP3 inhibitors may emerge as promising therapeutic agents for endothelial dysfunction and for reducing the burden of cardiovascular diseases.

### 7.2. Bottlenecks and Challenges in Clinical Translation

Although there are promising preclinical results, the clinical translation of NLRP3 inhibitors has been limited. A few compounds have progressed to clinical trials, including MCC950, OLT1177 (dapansutrile), and DFV890, which have been shown to possess anti-inflammatory and endothelial-protective properties [119,120,121]. In addition, chronic NLRP3 inhibition could also interfere with normal immune function and lead to heightened infection vulnerability [122]. Another challenge involves the pharmacokinetic limitations of several natural compounds, such as curcumin, resveratrol, and berberine. Although they have promising experimental efficacy, they may be less clinically effective due to poor bioavailability and rapid metabolism [123,124,125]. However, a number of issues still remain that need to be addressed to facilitate the clinical translation of NLRP3 inhibitors, such as long-term safety, risk of infection, and development of optimal dosing regimens. The potential for off-target effects and selectivity is incompletely understood, especially for compounds that are not extensively clinically evaluated. In addition, the ability to successfully target specific patients and diseases will be essential; therapeutic benefits of NLRP3 inhibition may differ between various cardiovascular and metabolic diseases [126,127]. Patient heterogeneity also complicates clinical application [128]. Since ED is found in a wide variety of diseases, such as diabetes, hypertension, atherosclerosis, and I/R injury, the therapeutic effects of NLRP3 inhibition may differ among patients and could be very different in those with different diseases [129].

Lastly, the majority of the available data for NLRP3 inhibitors comes from *in vitro* and animal research, and their efficacy and safety in humans are limited. Additional large-scale clinical studies are required to identify the best dosing plan and to assess the therapeutic potential of NLRP3 inhibitors in cardiovascular disease [122]. Overall, overcoming these translational barriers will be essential for the successful clinical implementation of NLRP3-targeted therapies.

## 8. Future Directions

### Emerging Approaches and Novel Therapeutics

Continuing the evolution of the concept of ED, the latest research points to the development of novel NLRP3-specific approaches, which have therapeutic possibilities. Natural products have proven to be of specific interest; an example is 13-MB, which stimulated autophagy in HUVECs and inhibited NLRP3 activation, whereas Aloe emodin (AE) prevented the effect of inflammasomes in C57BL/6J mice and EOMA cells [89,130]. Equally, the ROS/NLRP3 pathway activation due to high glucose concentration resulted in HMGB1 release, disruption of junctional protein (ZO-1/2), and endothelial hyperpermeability in diabetic C57BL/6J mice and cultured vascular endothelial cells. These effects were opposite and neutralized by silencing NLRP3 or inhibiting the ROS/HMGB1 axis, highlighting the mechanistic significance of the inflammasome regulation [75]. Future directions include individualized cardioprotection based on patient metabolic and immune characteristics, and particular emphasis on those groups at high risk, for whom IRI remains a significant clinical problem [131].

Encouraging results have also been recorded with pharmacological inhibitors. INF195 inhibited IL-β induced inflammation of the vascular endothelium *in vitro* in human macrophages and in *ex vivo* mouse hearts with I/R injury [34]. Another NLRP3-specific inhibitor, InflamAb, was important in reducing the endothelial inflammation in an experimental setting [118]. Interestingly, sex-specific differences in the benefit of vascular outcomes were found in a DOCA-salt rat model through NLRP3 inhibition, thereby suggesting the necessity to consider individualised treatment options [132]. In addition, MCC950 conserved the integrity of the blood–brain barrier of ischemic bEnd5 brain endothelial cells when used alongside rt-PA, indicating the feasibility of cerebrovascular use [133].

Collectively, these new methods highlight the heterogeneity of new inhibitors, including natural compounds as well as synthetic agents with uniform activity in endothelial and vascular systems. Translational research in the future must focus on closing these two gaps between preclinical observations and clinical development, to guarantee that effective inhibitors come out of the laboratory into the clinic in the management of ED and associated cardiovascular diseases.

Among the emerging therapeutic candidates, direct NLRP3 inhibitors such as MCC950 and OLT1177 appear particularly promising due to their well-characterized mechanisms of action, robust preclinical efficacy, and favorable translational potential. In contrast, newer agents, including INF195 and InflammAb require further validation regarding long-term safety, pharmacokinetic properties, and clinical applicability. With respect to disease models, diabetic vascular complications, atherosclerosis, and I/R injury represent highly relevant translational settings because ED is a central feature of these conditions, and substantial preclinical evidence already exists. Although combination therapy has not yet been demonstrated to be necessary, recent studies indicate that inflammatory activation of NLRP3 simultaneously with alternative disease mechanisms may optimize therapeutic responses. For instance, the synergistic effects of pelargonic acid vanillylamide (PAVA) and rosuvastatin against ED induced by ox-LDL were shown to be anti-NF-κB/NLRP3 signaling and anti-oxidative stress [134]. Likewise, co-inhibition of NLRP3 and activation of Amp-activated protein kinase (AMPK) with NLRP3 siRNA and 5-aminoimidazole-4-carboxamide ribonucleotide (AICAR) decreased inflammatory responses and restored endothelial function during metabolic stress conditions [135]. Furthermore, the dual-action berberine ursodeoxycholate (HTD1801) has demonstrated clinical effects in people with cardiometabolic diseases [136]. The concurrent use of NLRP3 inhibitors with established antidiabetic, antihypertensive, or lipid-lowering therapies may simultaneously target multiple pathological pathways involved in OxInflammation and ED, thereby enhancing therapeutic efficacy and improving vascular repair outcomes. Future studies should evaluate such combination strategies in clinically relevant disease models and patient populations.

## 9. Conclusions

ED is an initial cause of cardiovascular and metabolic syndromes, including atherosclerosis, hypertension, and diabetes mellitus. This is mediated by chronic low-grade inflammation, which is mostly driven by the NLRP3 inflammasome. In stressful conditions such as hyperglycemia or infection, the inflammasome facilitates the release of IL-1β and IL-18, which destroy the endothelium by raising leukocyte adhesion, increasing vascular permeability, and reducing the bioavailability of NO. Continuous stimulation of such a pathway maintains inflammation and promotes endothelial damage. Another potential way to interrupt such pathways is to target the NLRP3 inflammasome. Not only vascular inflammation reduced using inhibitors, but also the formation of atherosclerotic plaques and further cardiovascular incidents can be prevented. In addition to vascular health, inflammasome inhibition could lower systemic inflammation and oxidative stress, which cause insulin resistance, metabolic syndrome and even neuroinflammation in Alzheimer’s disease. Inflammatory pathways activated by these agents may have benefits in several comorbidities, providing benefits over disease-specific therapies. Clinical translation has its critical challenges despite the promising preclinical outcomes. The safety, specificity, and long-term efficacy of inflammasome inhibitors should be carefully evaluated in well-designed clinical studies. Special focus is required on the potential immune suppression, off-target effects, and identification of the optimal dosing, treatment period, and selection of patients. To enable targeted therapeutic interventions, comparative analyses across disease groups, such as diabetes and atherosclerosis, are required. In the future, the use of inflammasome inhibitors in practice will rely on the ability to show long-term cardiovascular and metabolic benefits and long-term safety. Combinations with current cardiovascular and metabolic therapy can also increase efficacy. Altogether, the inflammasome inhibitors may be considered a promising solution in the restoration of the endothelial health condition and minimizing the burden of chronic inflammatory diseases. Further studies and clinical validation will play an important role in establishing their role in disease prevention and treatment fully.

## Figures and Tables

**Table 1:** List of natural NLRP3 inhibitors and their effects on ED.

S. No	Name of the Compounds	Source	Experimental Model	Key Outcome	Mechanism in Endothelial Cells	Ref
1	Oridonin	*Rabdosia rubescens*	*In vitro*: Mouse peritoneal macrophages; *In vivo*: ApoE-deficient (ApoE ^−/−^) mice	Inhibited NLRP3; slowed atherosclerosis; mitigated inflammation and oxidative stress; preserved Nrf2 activity	Covalently binds to cysteine 279 of NLRP3; blocks NEK7 interaction and inflammasome assembly	[88]
2	Berberine	*Berberis* species	*In vitro*: Mouse microvascular endothelial cells (MECs); *In vivo*: LPS-administered mice	Re-established endothelial integrity through enhanced junction proteins, decreased inflammation, and reduced vascular injury via calcium influx regulation	Inhibits NLRP3 activation; restores junction proteins via calcium influx regulation	[87]
			*In vitro*: HUVECs	Inhibited endothelial inflammation and oxidative stress; restored nitric oxide bioavailability		[88]
3	Curcumin	*Curcuma longa*	*In vitro*: Human aortic endothelial cells (HAECs) *In vivo*: ApoE^−/−^ atherosclerotic mice	Enhanced endothelial function and reduced atherosclerosis-associated endothelial and inflammatory responses by inhibiting the activation of the NLRP3/ASC inflammasome	Blocks ASC speck formation and oligomerization; reduces IL-1β secretion	[93]
4	Resveratrol	Grapes, pine trees	*In vitro*: HUVECs *In vivo*: High-fat diet mice	Suppressed endothelial pyroptosis by regulating the SIRT1/p66Shc/NLRP3 pathway to restore endothelial integrity	Activates SIRT1; inhibits NF-κB/NLRP3/IL-1β signaling; suppresses pyroptosis	[96]
5	Naringenin	Citrus fruits	*In vitro*: Rat intestinal microvascular endothelial cells (RIMVECs)	Naringenin inhibited endothelial inflammation by blocking the hypersecretion of IL-6 and the activation of the TLR4/NF-κB/NLRP3	Suppresses NF-kB and NLRP3 inflammasome gene expression	[100]
6	Sinapine Thiocyanate (ST)	Seeds of cruciferous plants	*In vitro*: HUVECs exposed to Angiotensin II (AngII); *In vivo*: Spontaneously hypertensive rats (SHRs)	Preserved endothelial activity in hypertension by inhibiting inflammation and AngII-induced damage	Enhances NO production; reduces endothelin-1	[109]
7	Rutin	Citrus fruits, buckwheat	*In vitro*: HUVECs; *In vivo*: Sprague-Dawley rats	Preserved endothelial activity by inhibiting inflammation, oxidative stress, and NLRP3; re-established nitric oxide-mediated relaxation	Disturbs Nox4 and ROS-sensitive NLRP3 inflammasome; restores NO-mediated relaxation	[110]
8	Colchicine	Colchicum autumnale plant	*In vitro*: HUVECs; *In vivo*: Sprague-Dawley rats subjected to coronary microembolization (CME)	Inhibited inflammation and pyroptosis via the AMPK/SIRT1/NLRP3 pathway; blocked myocardial and endothelial injury	NLRP3 inhibition via AMPK/SIRT1 pathway	[111]

**Table 2:** Synthetic NLRP3 inflammasome inhibitors and their effects on ED.

S. No	Name of the Compounds	Experimental Models	Key Outcome	Ref
1	MCC950	*In vitro*: Human retinal endothelial cells (HRECs)	Reduced inflammation and cell death; preserved retinal endothelial integrity under high glucose conditions	[77]
		*In vitro*: Rat brain microvascular endothelial cells (BMVECs); *In vivo*: Male Wistar rats	Protected blood–brain barrier in diabetes and stroke; decreased inflammation; enhanced vascular and cognitive function	[76]
		*In vitro*: Hypoxia-induced THP-1 cells; *In vivo*: Oxygen-induced ischemic retinopathy (OIR) mouse model	Lowered IL-1β/IL-18; inhibited NLRP3; enhanced vascular integrity via reduced VEGF and MMPs	[112]
		*In vitro*: Murine macrophages and human coronary artery endothelial cells (hCAECs); *In vivo:* Mouse carotid interposition vascular graft model	Improved re-endothelialization; reduced vascular inflammation; preserved VE-cadherin and eNOS	[18]
2	CY-09	*In vivo*: ApoE-deficient (ApoE^−/−^) mice	Inhibited NLRP3 inflammasome; decreased aortic valve calcification and stenosis	[79]
3	OLT1177	*In vivo*: Murine model of myocardial ischemia–reperfusion injury (transient left coronary artery ligation)	Decreased infarct size, cardiac deficit, and myocardial harm; restricted inflammation	[83]
		*In vitro*: Human coronary artery endothelial cells (hCAECs); *In vivo*: C57BL/6 mice	Decreased inflammation and fibrosis; augmented endothelial activity; enhanced vascularization	[113]
		*In vitro*: Human microvascular endothelial cells (HMEC-1); *In vivo*: Wild-type and NLRP3 knockout C57BL/6J mice	Maintained endothelial integrity by inhibiting pyroptosis, IL-1β, and vascular leakage in DENV-induced dysfunction	[82]
4	3,4-Methylenedioxy-β-nitrostyrene (MNS)	*In vivo*: Rat model of ischemia–reperfusion injury	Suppressed tubular injury; inhibited PANoptosis	[86]
5	Glyburide	*In vitro*: high-salt-treated mouse aortic endothlial cells (MAECs) *In Vivo*: high-salt fed mice	Inhibits NALP3 signaling without involving the inflammasome, thus improving endothelial function, and restores the expression of p-eNOS	[104]
6	JC124	*In vitro*: Endothelial cells	Prevented the activation of the NLRP3 inflammasome and maintained the integrity of tight junctions, thus protecting against TMAO induced endothelial injury	[106]
7	Parthenolide	*In vivo*: LPS-induced sepsis in rats	Enhanced survival; improved mitochondrial integrity and endothelial barrier function	[108]
8	INF195	*In vitro*: LPS/ATP-stimulated human THP-1 macrophages; *Ex vivo*: Isolated mouse hearts subjected to 30 min global ischemia and 60 min reperfusion	Reduced pyroptosis and IL-1β levels; decreased infarct size and provided cardioprotection in I/R injury	[36]
		*In vitro*: Human coronary artery endothelial cells (HCAECs) and HUVECs exposed to oxidative stress (H_2_O_2_) and lipotoxic stress (palmitic acid)	Protected endothelial cells from oxidative and lipotoxic injury; reduced caspase-1, IL-1β, and pyroptosis; restored angiogenesis and endothelial function	[37]
9	INF150	*In vitro*: THP-1-derived macrophages and H9c2 cardiomyocytes; *Ex vivo*: Isolated male FVB mouse hearts subjected to I/R injury	Inhibited macrophage pyroptosis and IL-1β secretion	[36]
		*In vitro:* Human coronary artery endothelial cells (HCAECs) exposed to oxidative stress (H_2_O_2_) and lipotoxic stress (palmitic acid)	Reduced endothelial injury and inflammation, but showed lower efficacy than INF195 and required higher concentrations for protection	[37]
10	INF200	*In vitro*: Differentiated THP-1 macrophages stimulated with LPS/ATP and LPS/MSU; *In vivo*: High-fat diet (HFD)-induced male Wistar rats	Suppressed pyroptosis and IL-1β production, improved metabolic parameters, reduced inflammation, and provided cardioprotective effects in obese rats	[35]

**Table 3:** Summary of clinical trials and translational status of NLRP3 inhibitors.

S. No.	Inhibitor	Clinical Phase	Condition Studied	Key Clinical Findings	Safety/Tolerability	Study Limitations/Reason for Discontinuation	Development Status	References
1	MCC950	Preclinical/Early Phase I	Various inflammatory conditions	Potent NLRP3 inhibition; strong vascular protective effects in experimental models	Not evaluated in human studies	Evidence limited to in vitro and animal models; lack of clinical validation	Preclinical	[18,74,75,76,77]
2	CY-09	Preclinical	Atherosclerosis, diabetic nephropathy, inflammatory disorders	Reduced inflammation, oxidative stress, and endothelial injury in animal models	No major toxicity reported in preclinical studies	No human clinical trials; clinical safety and efficacy unknown	Preclinical	[64,79,117]
3	INF195	Preclinical	Myocardial I/R injury	Reduced IL-1β-mediated vascular inflammation and tissue injury	Effective at low doses in experimental models	Evidence limited to in vitro and ex vivo studies; absence of in vivo long-term safety and human clinical data	Preclinical	[34]
4	INF150	Preclinical	Cardioprotection in isolated mouse heart, macrophages, and H9c2 cardiomyocytes	Protected macrophages from LPS/ATP injury but did not enter H9c2 or differentiated H9c2 cells; no cardioprotective effect in isolated hearts, with infarct size, IL-1β, and cleaved caspase-1 unchanged	Appeared non-toxic in the tested macrophage/cellular settings, but functional cardiac benefit was absent	Failed to penetrate cardiomyocytes; ineffective in whole-heart model; no clinical phase data	Preclinical	[36]
5	InflammAb (bispecific antibody)	Preclinical	Atherosclerosis (ApoE^−/−^ mice)	Reduced endothelial inflammation and attenuated atherosclerotic progression	Not evaluated in human studies	No clinical studies; manufacturing and translational feasibility remain unknown	Preclinical	[118]
6	Berberine	Preclinical	Inflammatory vascular injury/endothelial junction dysfunction	Restored ZO-1 and VE-cadherin, inhibited endothelial NLRP3 activation, reduced TXNIP-NLRP3 binding, and suppressed ATP-induced Ca^2+^ influx in coronary microvascular endothelium and MECs	Not reported as a major issue in the study	Preclinical study only; no human clinical data	Preclinical	[88]

## Data Availability

No datasets were generated or analysed during the current study.
